# Self‐Injury and Domestic Violence in Young Adults During the COVID‐19 Pandemic: Trajectories, Precursors, and Correlates

**DOI:** 10.1111/jora.12659

**Published:** 2021-07-27

**Authors:** Annekatrin Steinhoff, Laura Bechtiger, Denis Ribeaud, Aja Louise Murray, Urs Hepp, Manuel Eisner, Lilly Shanahan

**Affiliations:** ^1^ Jacobs Center for Productive Youth Development University of Zurich; ^2^ Department of Psychology University of Edinburgh; ^3^ Integrated Psychiatric Services Winterthur‐Zürcher Unterland; ^4^ Institute of Criminology University of Cambridge; ^5^ Department of Psychology University of Zurich

**Keywords:** COVID‐19, self‐injury, domestic violence, young adulthood, longitudinal

## Abstract

We examined the longitudinal course of, and pre‐ and during‐pandemic risk factors for, self‐injury and domestic physical violence perpetration in young adults during the COVID‐19 pandemic. Data came from a Swiss longitudinal study (*N* = 786, age ˜22 in 2020), with one prepandemic (2018) and four during‐pandemic assessments (2020). The prevalence of self‐injury did not change between April (during the first Swiss national lockdown) and September 2020 (postlockdown). Domestic violence perpetration increased temporarily in males. Prepandemic self‐injury was a major risk factor for during‐pandemic self‐injury. Specific living arrangements, pandemic‐related stressor accumulation, and a lack of adaptive coping strategies were associated with during‐pandemic self‐injury and domestic violence. Stressor accumulation had indirect effects on self‐injury and domestic violence through negative emotions.

## INTRODUCTION

The transitions of adolescence and young adulthood are characterized by major changes (Arnett, 2000), including the developmental tasks of engaging in intimate relationships, gaining independence from the parental home (e.g., moving out), and laying the groundwork for educational and professional success. Tackling these tasks can be stressful, especially during a pandemic and its associated lockdowns. Indeed, the public health measures implemented to reduce the spread of coronavirus disease 2019 (COVID‐19), including social distancing, remote studying, and home‐office, run counter to the social nature of many of the activities typically embraced by young adults to achieve these developmental tasks. In addition, a pandemic compounds the normative transition stressors of young adults with additional new stressors. These include, for example, fewer social connections, uncertainty about the future, frustrations with society's response to the pandemic, and increased personal tensions in households (Brooks et al., 2020; Fegert, Vitiello, Plener, & Clemens, 2020; Shanahan et al., 2020).

Major stressors can trigger emotional dysregulation and subsequent maladaptive and harmful behaviors in some adolescents and young adults (Herts, McLaughlin, & Hatzenbuehler, 2012). Such harmful stress‐related behaviors include direct physical self‐injury with or without suicidal intent (e.g., self‐cutting; below referred to as self‐injury) (Brunner et al., 2014; Nock, 2009) and physical violence against others, such as household members (i.e., domestic violence perpetration) (Shorey, McNulty, Moore, & Stuart, 2015). Both affect a large percentage of adolescents and typically decline in young adulthood (Plener, Schumacher, Munz, & Groschwitz, 2015; Shulman, Steinberg, & Piquero, 2013; Steinhoff et al., 2021). These behaviors may, however, also (re)emerge and be triggered in the face of newly arising stressors.

The COVID‐19 pandemic and its associated lockdowns are among such potential stressors. Early on in the pandemic, scientific experts and public health officials raised concerns about the risk of increases in self‐injury and violence occurring in the home (Eisner & Nivette, 2020; Plener, 2021; Usher, Bhullar, Durkin, Gyamfi, & Jackson, 2020), but empirical work on this topic in young adults has not followed suit. Large‐scale community data are needed to understand the longitudinal trajectory of self‐injury and domestic physical violence perpetration across different phases of the pandemic, including the during‐ and postlockdown phases, which may be associated with differential stressor loads.

A better understanding of the individual risk factors that increase young people's likelihood of engaging in self‐injury and domestic violence during pandemics is also needed. Relevant risk factors most likely include prepandemic factors (e.g., prepandemic self‐injury or violence perpetration) and during‐pandemic factors, such as pandemic‐related stressor accumulation and maladaptive coping with the pandemic (e.g., nonacceptance). Finally, knowledge about the emotional and behavioral mechanisms through which pandemic‐related stressors increase individuals' risk of self‐injury and domestic violence perpetration is needed. This will help develop informed interventions and support individuals at risk during and after this and future pandemics.

### Risk Factors

#### Challenges of emerging adulthood: Living arrangements

Due to stay‐at‐home orders during lockdowns, and social distancing measures, the pandemic has shone the spotlight on living arrangements and domestic relationships as important contexts for well‐being. Gaining residential independence (e.g., moving out of the parental home to cohabit with a romantic partner or peers or to live alone) is traditionally considered a transition marker of young adulthood, typically achieved at some point during one's early twenties (Seiffge‐Krenke, 2009), although this milestone has tended to be delayed in recent years due to economic reasons (Berngruber, 2015). During the pandemic, moving out of the parental home may be further complicated and being forced to stay in the parental home while striving for independence may be a stressor in itself. Accordingly, interpersonal tensions in the household could increase, which, in turn, could trigger self‐injury and domestic violence.

Cohabiting with parents could, however, also be a protective during‐pandemic factor. Previous research has shown that young adults with mental health problems who still live with parents have a higher likelihood of receiving adequate mental health care compared with those who have moved out of the parental home (Copeland et al., 2015). These differences may be exacerbated during lockdowns when psychological services may be more difficult to access (Hawton et al., 2021).

For young adults living alone, lockdowns may increase social isolation and loneliness, which are risk factors for self‐injury (Hawton et al., 2021; Shaw et al., 2021). By contrast, living with (supportive) others (e.g., parents, peers, or intimate partners) during a pandemic may be protective because these relationships are typically important sources of social support, which counteracts psychological problems (Finan, Ohannessian, & Gordon, 2018).

Some young adults have moved in with a romantic partner before the pandemic, which is a major challenge in and of itself (S. Shulman & Connolly, 2013). During the pandemic, this normative transition‐related stressor was likely compounded for many couples by the need to navigate new pandemic‐related stressors, increased time together, decreased social time with others, fewer routines and less structure in daily life, and less freedom to spend time apart or to separate. This combination of pressures can place serious strain on a relationship, which, in turn, increases the risk of domestic violence (O'Leary, Tintle, & Bromet, 2014).

#### Prepandemic harm

Although the pandemic creates new and exceptional situations for young people, harmful behaviors during this period are likely in part a function of prepandemic behavioral development. There is strong evidence of longitudinal recurrence of adolescent violent behaviors, including self‐injury (Moran et al., 2012; Plener et al., 2015) and dating violence (Fernandez‐Gonzalez, Calvete, & Orue, 2020). Thus, having a prior history of self‐injury or violence perpetration is likely to be a major risk factor for the maintenance or relapse of these behaviors during times of increased stress, such as during a pandemic.

#### During‐pandemic stressors and coping

Acute stressors (e.g., interpersonal distress) and their accumulation play an important role in triggering self‐injury (Hooley & Franklin, 2017) and violence against others (Chen & Foshee, 2015). In a prior study based on the same sample used here, we found that some secondary consequences of the pandemic (e.g., economic disruptions) increased the risk of young adults' perceived stress, internalizing symptoms, and anger (Shanahan et al., 2020). While the experience of one stressor at a time may be relatively manageable, an accumulation of stressors is likely to become so stressful that engagement in maladaptive behaviors is increasingly likely (Appleyard, Egeland, Van Dulmen, & Sroufe, 2005; Steinhoff, Bechtiger, Ribeaud, Eisner, & Shanahan, 2020). However, the associations of (1) cumulative pandemic‐related stressors (e.g., exposure to the disease, financial difficulties, disruptions of work and educational arrangements) and (2) prepandemic stressors (e.g., stressful life events) with during‐pandemic self‐injury and domestic violence have not been examined.

A lack of adaptive coping strategies could also increase the risk of self‐injury (Guerreiro et al., 2013) and physical aggression (Whitman & Gottdiener, 2015) and thus domestic violence perpetration during the pandemic. For example, maladaptive emotion‐focused strategies, such as avoidance, have been linked to self‐injury (Anderson & Crowther, 2012) and physical aggression (Carlo et al., 2012). In fact, successful treatment of self‐injury often involves fostering acceptance of unpleasant circumstances or feelings (Nock, Teper, & Hollander, 2007) rather than avoiding them. Additional adaptive coping strategies, including cognitive reappraisal and reframing processes (e.g., finding something good in the current situation), tend to be effective in the treatment of depression and anxiety, both of which are closely related to self‐injury and domestic violence perpetration (Butler, Chapman, Forman, & Beck, 2006; Troy, Wilhelm, Shallcross, & Mauss, 2010). Finally, nonsuicidal self‐injury is sometimes used as a means to distract from negative emotions and rumination (Selby, Franklin, Carson‐Wong, & Rizvi, 2013). Thus, infrequent use of adaptive strategies to distract oneself from acute stress could be associated with an increased risk of self‐injury and perhaps also domestic violence perpetration.

#### Mechanisms that translate pandemic‐related stressors into harm

Emotional dysregulation plays an important role in both self‐injury and violence perpetration (Nock, 2009; Shorey et al., 2015). Accordingly, strong negative emotions, such as depressive and anxiety symptoms and anger, may be involved in translating pandemic‐related exposure to stressors into these behaviors (Farrington, 2018; Laye‐Gindhu & Schonert‐Reichl, 2005; Nock, Joiner, Gordon, Lloyd‐Richardson, & Prinstein, 2006). However, these mechanisms have not been examined in the context of the COVID‐19 pandemic.

### The Current Study and Its National Context

The current study leverages data from a prospective longitudinal community study to investigate (1) the average longitudinal course of self‐injury and domestic physical violence perpetration in young adults during and after the first Swiss COVID‐19 lockdown period, (2) the prepandemic and during‐pandemic risk factors for these behaviors, and (3) the emotional mechanisms involved in the pathways from pandemic‐related stressor accumulation to self‐injury and domestic violence perpetration. We hypothesized (1) an increased risk of self‐injury among those living alone compared with those living with parents, peers, or an intimate partner, as well as an increased risk of domestic violence perpetration in households with intimate relationships, including with a partner or parents, compared with households with peer‐relationships. We further hypothesized that (2) prepandemic histories of self‐injury and violence perpetration increase the risk of during‐pandemic self‐injury and domestic violence, respectively; and (3) pandemic‐related stressor accumulation and lack of adaptive coping strategies are associated with an increased risk of both outcomes. Although the important role of adaptive coping in dealing with daily strains has been documented in prior research, pandemic‐related coping in relation to self‐injury and domestic violence perpetration has not been investigated previously. Therefore, we explored the role of different strategies (e.g., nonacceptance of the pandemic, reappraisal, distraction, and several others). Finally, we explored whether pandemic‐related stressor load was linked to subsequent self‐injury and domestic violence perpetration through internalizing symptoms and anger.

**FIGURE 1:**
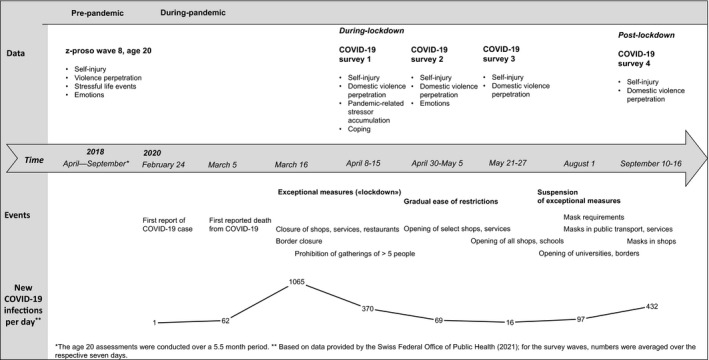
Study Design in the Context of the Chronicles of the COVID‐19 Pandemic and Associated Regulations in Switzerland in 2020.

Figure 1 presents our study design in the context of the pandemic and associated regulations in Switzerland, which has a population size of ˜8.6 million. Cases of COVID‐19 infections increased in spring 2020. On March 16th, the government imposed ‘exceptional measures’, which ushered in what we call the ‘lockdown’ period, including closures of schools, universities, shops, and borders to several neighboring countries, and orders for home working whenever possible (Kohler, Hauri, Scharte, Thiel, & Wenger, 2020). Regulations were gradually loosened beginning in late April. The re‐opening of select shops started on April 27th. Educational institutions were allowed to reopen between mid‐May (e.g., primary schools) and June (e.g., secondary schools), although many institutions (e.g., universities) continued to offer remote instruction only. The ‘exceptional’ state was suspended on June 19, 2020, when reported new cases of COVID‐19 infections in Switzerland had diminished to 10–30 per day (Federal Office of Public Health Switzerland, 2021). Many people were able to return to an almost normal life domestically (e.g., go back to work, at least part‐time). Rules for social distancing and the prohibition of major events were still in place, and as of July 6th, wearing a mask in public transport was required. Our study includes one prepandemic assessment (in 2018) and four repeated during‐pandemic assessments. One was in early April (reflecting week 4 of the strictest lockdown measures), the second in early May, the third in late May, and the fourth in September (postlockdown and before the ‘second wave’ of COVID‐19 infections, which accelerated in October). This study design uniquely positioned us to assess pre‐ and during‐pandemic risk factors and during‐ and postlockdown self‐injury and domestic violence perpetration.

## METHODS

### Participants

We used data from the Swiss longitudinal community‐representative Zurich Project on the Social Development from Childhood to Adulthood (z‐proso) (Ribeaud & Eisner, 2010). The initial target sample included 1675 children who entered 1st grade in one of 56 public primary schools in Zurich (Switzerland's largest city) in 2004. The schools were selected using random sampling procedures, with slight oversampling of disadvantaged school districts. Participants were assessed eight times between ages 7 (in 2004; *n* = 1360) and 20 (in 2018; *n* = 1180). Consistent with Zurich's diverse population, the parents of the participants had been born in >80 different countries. Those who participated in 2018 were subsequently invited to participate in four additional waves of data collection between April and September 2020. At that time, participants were ˜22 years old. Here, we use data from the 2018 assessment (i.e., prepandemic) and the four 2020 (i.e., during‐pandemic) assessments. We include all respondents who participated in at least the April 2020 assessment (*n* = 786, 58.3% female).

### Procedures

In 2018, participants completed interview surveys (typically lasting ˜70 min) at a university research laboratory. In 2020, four online surveys (lasting ˜15–20 min to complete) were conducted; participants were given 7 days to complete each survey. In 2018, respondents received a ˜$75 cash incentive; in 2020, respondents were entered into a lottery to win one of 50 prizes of ˜$100. Participants provided written informed consent for their study participation at ages 13‐20 (parents of children below age 15 could opt their child out of the study), and online informed consent at age 22. Ethical approval was obtained from the Ethics Committee of the Faculty of Arts and Social Sciences of the University of Zurich. All procedures contributing to this work comply with the ethical standards of the relevant national and institutional committees on human experimentation and with the Helsinki Declaration of 1975, as revised in 2008.

### Variables

#### During‐pandemic outcomes

*Self‐injury* was assessed at each of the four COVID‐19 assessments, with one item that asked how often respondents had self‐injured (e.g., cutting, tearing wounds open, hitting one's head, tearing out one's hair) on purpose during the previous 2 weeks (Steinhoff et al., 2021). This broad assessment excluded self‐poisoning, did not distinguish between particular motivations or severity of the injury, and could include both suicidal and nonsuicidal self‐injury. Answers were recorded on a five‐point scale (1 = never, 2 = rarely, 3 = sometimes, 4 = often, 5 = very often) and then dichotomized, to assess whether participants had engaged in any self‐injury (i.e., at least rarely = at least once).

*Perpetration of domestic physical violence* was also assessed at all four assessments in 2020. From six items assessed, which were adapted from the Conflict Tactics Scale (Straus, 1979) and relate to both physical and verbal violence, we selected three that addressed physical violence (Cronbach's α = .76, .65, .70, and .84 for the four assessments). Items asked how often participants had attacked (e.g., slapped or scratched) household members during the previous 2 weeks (1 = never, 2 = once, 3 = 2–4 times, 4 = 6–10 times, 5 = more than 10 times). To differentiate individuals who engaged in any domestic physical violence versus those who did not, we created a dichotomous variable indicating at least one incidence of physical violence.

#### During‐pandemic correlates

*Living arrangements* were assessed in April 2020. The respondents reported whether they currently lived with their parents (including biological parents and step‐parents), with an intimate partner, in a shared apartment with other peers, or alone.

*Cumulative stressors* were also assessed in April 2020. The participants were asked whether they had experienced a set of events since the beginning of the pandemic (i.e., during the previous ˜2 months). We selected 15 potentially stressful events, including health‐related (e.g., exposure to COVID‐19) and economic and educational events (e.g., job loss) and events that happened to the participants or their close others (e.g., parents). A full list of items is provided in the online supplement (Table S1). For each event, participants were assigned 1 if an event had occurred (or 0 otherwise), and the items were summed. Overall, 55% of participants reported at least one stressor.

To assess *infrequent use of adaptive coping strategies,* in April 2020, participants were asked to indicate how often they engaged in particular activities when having experienced something stressful during the previous 2 weeks (four‐point scale: 1 = never, 4 = very often). Each strategy was assessed with one item, adapted from Carver (1997), indicating acceptance of the corona crisis as something real, trying to find something good in the corona crisis (i.e., positive reappraisal), self‐distraction, emotional support seeking, and keeping in contact with close others. Additional strategies assessed were keeping a daily routine and physical exercise. Details on item formulations are provided in the online supplement (Table S2). Responses were re‐coded, with higher values representing a lower frequency of using the respective strategy.

#### Mediators

*Internalizing symptoms* in early May 2020 were assessed with 13 items from the Social Behavior Questionnaire (SBQ; Murray, Obsuth, Eisner, & Ribeaud, 2019; Tremblay et al., 1991) that assessed how often participants cried, worried, and felt frustrated, scared, unhappy, alone, and sad without reasons, had negative thoughts about themselves and sleep problems during the previous 2 weeks (five‐point scale: 1 = never, 5 = very often). A mean score was created with higher values indicating more symptoms (α = .91).

*Anger* was also assessed in early May 2020, with three items from the PROMIS Emotional Distress—Anger—Short Form (Pilkonis et al., 2011) asking how often participants had feelings of anger, frustration, or annoyance during the previous 2 weeks (five‐point scale: 1 = never, 5 = very often). A mean score was created with higher values indicating more anger (α = .81).

#### Prepandemic precursors

In 2018, *self‐injury* was assessed with the same item used during the pandemic, except participants were asked to refer to the previous month (instead of the previous 2 weeks). As with the during‐pandemic variable, we created a dichotomous indicator of whether the respondents had engaged in self‐injury at least once during the past month.

To assess prepandemic *perpetration of violence*, two measures were used. First, *physical dating violence* was assessed with the same items used to assess domestic physical violence during the pandemic (α = .69). The items referred to dating violence instead of violence toward a household member, and the reference period was the previous 12 months. Response options were 1 = never, 2 = 1–3 times, 3 = 4–9 times, 4 = more than 9 times. We also included a more *generic indicator of violence perpetration*, combining two items, one from the SBQ (Tremblay et al., 1991), asking how often during the previous year the respondents had hit, bitten, kicked, or pulled the hair of somebody else (six‐point scale: 1 = never, 6 = [almost] daily), and one from a delinquency scale designed by the project team, asking whether respondents had hurt somebody else by hitting, kicking, or cutting them on purpose (yes or no). For both, dating violence and any violence, we created dichotomous variables with 1 = any engagement in the behavior.

*Cumulative stressful life events* were assessed in 2018 by asking the respondents to report on the occurrence of 28 potentially stressful events since the previous assessment (at age 17). The list of events included in this score is reported in the online supplement (Table S1). We created a sum score of the number of events reported, to capture the overall stressor load from life events. *Internalizing symptoms* (α = .92) and *anger* (α = .71) were also assessed in 2018, with the same items used in the during‐pandemic assessment.

#### Control variables

Control variables were respondents' *sex* (1 = female, 0 = male), *parental socio‐economic status* (SES) during the respondents' adolescence (measured as International Socio‐Economic Index [Ganzeboom, De Graaf, Treiman, & De Leeuw, 1992] on a scale from 16 to 90, which was reverse coded for the main analyses, so that higher values indicate lower SES), and *parental migration background* (1 = both parents born abroad, 0 = at least one parent born in Switzerland).

### Analytic Strategy

First, we compared the prevalence of self‐injury and domestic violence perpetration across the four assessments in 2020 (i.e., during the ‘lockdown’ in April, the gradual loosening of measures in early and late May, and postlockdown, in September), conducting descriptive analyses using IBM SPSS V25. Second, longitudinal models were specified using Mplus V8. We estimated differences in individuals' risk of engaging in self‐injury and domestic physical violence during different phases of the pandemic using two‐level random intercept models (Hoffmann, 2014). These models account for the nesting of repeated assessments over time within individuals. The phases of the pandemic (i.e., during‐lockdown, intermediate, and postlockdown) were specified as categorical time‐varying predictors at the within‐person level and odds ratios (OR) from logistic regression analyses are provided.

In a second set of models, we examined risk factors for self‐injury and domestic violence occurring across the 2020 study period. To consider all available data and account for their nested structure, we also specified multilevel models, with risk factors included as time‐invariant (between‐person level) predictors of the individuals' random intercepts of self‐injury and domestic violence perpetration. The latter were modeled as continuous latent variables, representing an individual's propensity to engage in self‐injury or domestic violence, respectively, across the four assessments. Multivariable models included sociodemographics and all predictors for which hypotheses were formulated (i.e., living arrangements, prepandemic self‐injury or violence, during‐lockdown stressor accumulation), plus predictors that were explored without having specific hypotheses (i.e., prepandemic stressful life events, emotions, specific coping strategies) and that were significantly associated with the respective outcome on the bivariate level. Maximum‐likelihood estimation with robust standard errors (MLR) was used.

Third, we specified models estimating direct and indirect paths within a structural equation modeling framework (Hayes, 2009) to assess indirect effects from stressor accumulation by April 2020 to subsequent self‐injury and domestic violence, reported in late May and September, via emotions reported in early May (see supplementary Figure S1 for a path diagram). The MPlus MODEL INDIRECT command and maximum‐likelihood estimation were used, and bias‐corrected bootstrapped standard errors (1000 draws) were computed to detect significant effects (MacKinnon, Lockwood, & Williams, 2004).

### Handling of Missing Data due to Sample Attrition and Selectivity

Details on sample attrition in z‐proso until April 2020 can be found elsewhere (Eisner, Murray, Eisner, & Ribeaud, 2018; Shanahan et al., 2020). Here, we compare respondents who participated in April 2020 only to those who participated in all four COVID‐19 assessments. Overall, 67% (*n* = 525) of those who participated in April participated until September. Attrition was not related to prepandemic self‐injury and violence perpetration, or self‐injury, domestic violence, and accumulated pandemic‐related stressors reported in April 2020. Males were slightly more likely than females to drop out of the COVID assessments (36.6% vs. 30.8%, *p* = .089). Those whose parents were both born abroad were more likely to drop out than those with at least one parent born in Switzerland (41% vs. 27%, *p* < .001). September participants had a higher SES than those who no longer participated (*M* = 49.66, SD = 19.74 vs. *M* = 46.22, SD = 19.59, *p* = .024). Little's MCAR test was significant (*p* < .001), indicating that data were not missing completely at random, but either missing at random or not at random (see e.g., Allison, 2001).

To generalize back to the original community‐representative recruitment population, we used sampling weights. The weights take into account participation probabilities based on sex and migration background (for details, see Nivette et al. (2021)). In the descriptive analyses, we use weights calculated for each of the four during‐pandemic assessments separately. In the longitudinal models, we use weights for the first during‐pandemic assessment (on the between‐person level), and full‐information maximum likelihood (FIML) to account for missing data due to sample attrition that occurred thereafter (Enders, 2013; Schafer & Graham, 2002).

## RESULTS

Table 1 shows the descriptive statistics for all predictor and potential mediator variables in the unweighted and weighted sample. The distribution of living arrangements in our sample is typical for this age‐group in Zurich, where expensive housing precludes many young adults from leaving the parental home.

**TABLE 1:** Descriptive Sample Statistics for Potential Correlates of Self‐Injury and Domestic Violence Perpetration

	Unweighted sample		Weighted sample	
	% (*n*)	*M* (SD)	% (*n*)	*M* (SD)
Sociodemographics				
Sex (female)	58.3 (458/786)		48.1 (378/786)	
Low SES		41.49 (19.74)		43.05 (20.02)
Parental migration background	45.0 (348/774)		50.9 (394/775)	
Living situation				
With parents	69.2 (544/786)		71.6 (562/786)	
With peers	13.7 (108/786)		12.6 (99/786)	
With partner	9.8 (77/786)		8.7 (69/786)	
Alone	5.5 (43/786)		5.3 (42/786)	
Prepandemic				
Stressful life events		3.59 (2.35)		3.60 (2.36)
Internalizing symptoms		2.30 (0.81)		2.61 (0.80)
Anger		2.41 (0.76)		2.37 (0.75)
Self‐injury	7.3 (57/786)		6.7 (53/786)	
Dating violence	14.2 (111/783)		14.8 (116/784)	
Any violence	5.5 (43/784)		5.9 (47/784)	
During lockdown (April)				
Cumulative stressors		0.92 (1.11)		0.92 (1.13)
Nonacceptance		1.84 (0.80)		1.86 (0.83)
Low cognitive reappraisal		2.33 (1.03)		2.34 (1.04)
Low self‐distraction		2.07 (0.87)		2.10 (0.88)
Low emotional support seeking		2.87 (0.90)		2.93 (0.89)
Infrequent social contacts		2.00 (0.83)		2.02 (0.83)
Infrequent physical exercise		2.43 (1.05)		2.46 (1.04)
Lack of daily routine		2.21 (0.90)		2.24 (0.90)
Late May				
Internalizing symptoms		2.20 (0.79)		2.17 (0.78)
Anger		2.62 (0.94)		2.58 (0.94)

### Longitudinal Course of Self‐injury and Domestic Physical Violence Perpetration During the Pandemic

**FIGURE 2:**
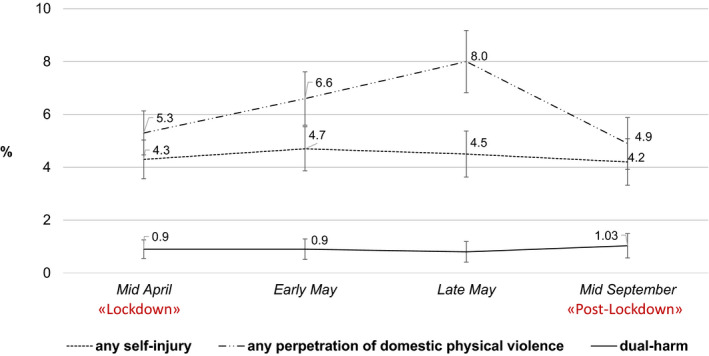
Longitudinal Course of Self‐Injury, Domestic Physical Violence Perpetration, and the Co‐Occurrence of Both Behaviors (i.e., Dual‐Harm) Among Young Adults Between Spring and Fall 2020. *Note*. Based on weighted sample. The exact prevalence of dual‐harm in late May is not provided to avoid reporting potentially identifying information due to a very small cell size (*n* < 5). Prevalence of domestic violence perpetration and dual‐harm refers to the group of participants who were not living alone.

Overall, 9% of the participants reported any self‐injury at one or more assessments between spring and fall 2020; 16% of those not living alone reported any perpetration of domestic physical violence. A total of 3% reported both self‐injury and domestic violence (i.e., dual‐harm) either at the same or at different assessments. Figure 2 shows the prevalence of any self‐injury and domestic violence perpetration across the four assessments. According to the multilevel models, the risk of self‐injury did not change during different phases of the pandemic; the risk of domestic violence perpetration was marginally higher in late May (after lockdown measures had been in place for almost 2 months and were now gradually loosened) compared to late April (the first lockdown; Table 2). Additional sex‐specific models revealed an increase of domestic violence perpetration among males, from 5% in April to 10% in late May (*p* = .014), but not females (see supplementary Figure S2). The prevalence of dual‐harm among those not living alone was ˜1% at all assessments.

**TABLE 2:** Associations Between Risk Factors and Self‐Injury in 2020, Fixed Effects From Multi‐Level Regression Models

		During‐pandemic self‐injury						During‐pandemic domestic physical violence perpetration					
		Unadjusted			Multivariable model^++^			Unadjusted			Multivariable model^++^		
*Within‐level*		**OR**	**95%CI**	***p***				**OR**	**95%CI**	***p***			
Phase of pandemic	Ref.												
Early May	Lockdown (April)	1.24	0.64 to 2.39	.530				1.28	0.75 to 2.20	.332			
Late May		1.25	0.65 to 2.42	.506				***1.70***	0.93 to 3.08	.086			
September (postlockdown)		1.17	0.57 to 2.40	.670				0.85	0.42 to 1.74	.665			
*Between‐level*		***β***	**95%CI**	***p***	***β***	**95%CI**	***p***	***β***	**95%CI**	***p***	***β***	**95%CI**	***p***
Sociodemographics													
Sex (female)		−0.03	−0.18 to 0.12	.685	−0.13	−0.29 to 0.03	.117	−0.02	−0.16 to 0.13	.844	−0.11	−0.28 to 0.05	.162
Low SES		0.06	−0.08 to 0.20	.395	−0.01	−0.18 to 0.16	.907	**0.23**	0.07 to 0.39	.006	**0.18**	0.01 to 0.34	.034
Migration background		0.06	−0.09 to 0.21	.453	0.03	−0.15 to 0.20	.781	**0.16**	0.01 to 0.30	.036	−0.01	−0.16 to 0.15	.935
Living arrangements	Ref.												
With peers	Parents	0.09	−0.05 to 0.22	.231	0.04	−0.11 to 0.19	.579	−0.11	−0.27 to 0.06	.214	−0.13	−0.30 to 0.04	.137
With partner		0.09	−0.06 to 0.23	.227	0.07	−0.10 to 0.25	.418	***0.11***	−0.02 to 0.23	.086	0.10	−0.03 to 0.23	.142
Alone		**0.20**	0.08 to 0.31	.001	**0.16**	0.06 to 0.27	.002	—	—	—	—	—	—
With peers	Partner	−0.02	−0.22 to 0.18	.850	−0.04	−0.27 to 0.19	.722	−**0.23**	−0.43 to −0.03	.027	−**0.25**	−0.45 to −0.04	.021
Alone		0.12	−0.04 to 0.28	.127	0.11	−0.06 to 0.27	.205	—	—	—	—	—	—
Alone	Peers	***0.14***	−0.00 to 0.28	.056	***0.14***	−0.00 to 0.27	.053	—	—	—	—	—	—
Prepandemic													
Stressful life events		**0.17**	0.05 to 0.29	.006	0.05	−0.10 to 0.20	.516	**0.22**	0.11 to 0.34	<.001	***0.12***	−0.02 to 0.26	.088
Internalizing symptoms		**0.21**	0.07 to 0.35	.003	0.07	−0.16 to 0.30	.559	**0.15**	0.02 to 0.28	.028	0.01	−0.19 to 0.20	.953
Anger		**0.19**	0.03 to 0.35	.021	0.00	−0.21 to 0.22	.985	**0.20**	0.07 to 0.33	.003	**0.20**	0.02 to 0.39	.033
Self‐injury		**0.31**	0.21 to 0.40	<.001	**0.27**	0.16 to 0.39	<.001	—	—	—	—	—	—
Dating violence		—	—	—	—	—	—	0.09	−0.04 to 0.22	.157	—^a^	—	—
Any violence perpetration		—	—	—	—	—	—	**0.17**	0.05 to 0.30	.008	0.09	−0.03 to 0.21	.138
During‐lockdown (April)													
Cumulative stressors		**0.24**	0.09 to 0.39	.002	**0.17**	0.02 to 0.32	.026	**0.19**	0.06 to 0.33	.006	**0.16**	0.03 to 0.29	.018
Nonacceptance		**0.23**	0.09 to 0.37	.001	**0.20**	0.06 to 0.34	.005	**0.14**	0.01 to 0.28	.032	**0.16**	0.03 to 0.29	.015
Low cognitive reappraisal		**0.15**	0.00 to 0.29	.044	0.03	−0.12 to 0.17	.703	0.07	−0.08 to 0.21	.368	—^b^	—	—
Low self‐distraction		0.07	−0.08 to 0.21	.366	—^b^	—	—	−0.11	−0.25 to 0.03	.129	—^b^	—	—
Low emotion. support		−0.06	−0.20 to 0.08	.421	—^b^	—	—	0.01	−0.13 to 0.15	.896	—^b^	—	—
Infreq. social contacts		***0.13***	−0.02 to 0.27	.079	−0.06	−0.22 to 0.09	.430	−0.02	0.17 to 0.13	.795	—^b^	—	—
Infreq. physical exercise		***0.14***	−0.01 to 0.29	.060	0.03	−0.14 to 0.20	.739	***0.12***	−0.02 to 0.26	.099	0.04	−0.11 to 0.19	.615
Lack of daily routine		**0.20**	0.07 to 0.33	.003	0.04	−0.11 to 0.19	.624	0.06	−0.08 to 0.19	.415	—^b^	—	—

Note

^++^self‐injury: *n*(subjects) = 728, *n*(observations) = 2345; domestic violence: *n*(subjects) = 684, *n*(observations) = 2204; ^a^excluded because of conceptual overlap with generic violence, which is broader and significantly correlated with the outcome on the bivariate level; ^b^excluded because no specific hypotheses were formulated and bivariate associations were nonsignificant.

Within‐level: logistic regression, between‐level: linear regression (random intercept is continuous latent variable); bold print indicates significant effects (*p* ≤ .05), bold and italic indicates trends (*p* ≤ .10).

### Precursors and Correlates of During‐Pandemic Self‐Injury

Unadjusted coefficients show that the risk of self‐injury was higher among those living alone compared to those cohabiting with parents or peers, but not compared to those living with an intimate partner (Table 2). Prepandemic stressful life events, internalizing symptoms, anger, and a history of prior self‐injury were significant antecedents of during‐pandemic self‐injury. Pandemic‐related cumulative stressors and low levels of several coping strategies were also associated with self‐injury. The multivariable models revealed unique associations of during‐pandemic self‐injury with living alone, a prior history of self‐injury (this was the strongest correlate), cumulative pandemic‐related stressors, and nonacceptance of the pandemic.

### Precursors and Correlates of During‐Pandemic Domestic Physical Violence Perpetration

Unadjusted coefficients show that low SES and parental migration background were associated with a higher risk of perpetrating domestic physical violence during the pandemic (Table 2), but the latter association was nonsignificant when both of these variables were simultaneously included in one model. Young adults cohabiting with an intimate partner had a higher risk of domestic violence perpetration than those living with peers, and, at the trend level, those living with parents. Prepandemic stressful life events, internalizing symptoms, anger, and a history of generic violence perpetration were antecedents of during‐pandemic domestic violence. Cumulative pandemic‐related stressors and select coping strategies were also associated with domestic violence. The multivariable models indicated unique associations of domestic violence perpetration with low SES, cohabiting with a partner versus peers (this factor had the strongest association with domestic violence), prepandemic anger, during‐pandemic stressor accumulation, and nonacceptance of the pandemic.

### Indirect Effects

The models of indirect effects from during‐lockdown stressor accumulation to self‐injury in late May and in September revealed a significant role of negative emotions, including both internalizing symptoms and anger (Table 3). Internalizing symptoms were also involved in the chain from stressors accumulation to domestic physical violence perpetration.

**TABLE 3:** Direct and Indirect Effects of Pandemic‐Related Cumulative Stressors During the Lockdown (April) on Self‐Injury and Domestic Violence Perpetration in Late May and September 2020

Paths	Self‐injury				Domestic physical violence perpetration			
	Late May (third month of exceptional measures)		September (postlockdown)		Late May (third month of exceptional measures)		September (postlockdown)	
	*b* ^+^	95% CI^++^	*b* ^+^	95% CI^++^	*b* ^+^	95% CI^++^	*b* ^+^	95% CI^++^
Cumulative stressors → internalizing symptoms								
Direct effect of cumulative stressors	0.15	−0.49 to 0.55	0.21	−0.58 to 0.65	0.14	−0.33 to 0.55	0.33	−0.26 to 0.71
Indirect effect through internalizing symptoms	**0.06**	0.01 to 0.14	**0.05**	0.00^a^ to 0.13	**0.04**	0.00^a^ to 0.10	**0.05**	0.00^a^ to 0.14
Cumulative stressors → anger								
Direct effect of cumulative stressors	0.18	−0.45 to 0.56	0.22	−0.56 to 0.65	0.19	−0.26 to 0.58	0.32	−0.36 to 0.71
Indirect effect through anger	**0.05**	0.00^a^ to 0.13	**0.06**	0.00^a^ to 0.15	0.03	−0.00 to 0.08	0.06	−0.00 to 0.19

Note

^+^unstandardized coefficient; **^++^**bootstrapped, 1000 draws.

For these analyses, the use of sampling weights was not available. Models controlled for sociodemographics, prepandemic self‐injury/violence perpetration, respectively, prepandemic internalizing symptoms/anger, respectively, and living arrangements (see online supplement for a graphical illustration of the model specification). Bold print indicates significant effects; ^a^the lower level of these coefficients was >0.00 and appears as .00 in the table due to rounding).

## DISCUSSION

Transitioning to young adulthood brings major challenges for young people. Our study provides novel insights into how a major stressor, such as the COVID‐19 pandemic, can compound these challenges and for whom. In our Swiss community sample, the prevalence of self‐injury did not change significantly between April (the first national lockdown) and September 2020 (when lockdown measures had been lifted and reported cases of COVID‐19 infections were at low levels). However, a significant temporary increase in domestic violence perpetration among males during this period underscores the importance of understanding how the pandemic and its associated restrictions are linked with individual differences in mental health and behavioral development. Our findings revealed associations between individual stressor accumulation during a lockdown period and a lack of specific adaptive coping strategies with both self‐injury and domestic physical violence perpetration across the first half‐year of the pandemic. Risk factors partly differed for self‐injury versus domestic violence, which has implications for intervention practices during and after the pandemic.

### The Longitudinal Course of Self‐Injury and Domestic Violence Perpetration During the Pandemic

Although our data suggest that, on average, the prevalence of self‐injury did not increase during the first months of the pandemic in Switzerland, it is possible that pandemic‐related stressors prevented an otherwise normative age‐related decrease of this behavior during young adulthood (Plener et al., 2015; Steinhoff et al., 2021) in some individuals. Indeed, we found that the individual risk of self‐injury was associated with several individual pandemic‐related risk factors.

The finding that domestic physical violence perpetration increased in males during a period when exceptional governmental restrictions were in force confirms the concerns that several researchers and public health officials raised early on during the COVID‐19 pandemic (Usher et al., 2020). Risk factors for this sex‐specific trend need to be investigated in future studies to prevent the various long‐term sequelae of domestic physical violence (Kofman & Garfin, 2020; Stewart & Robinson, 1998). One possible explanation could be that males have a lower threshold for resorting to violence than females when feeling frustrated.

### Living Arrangements

The findings are consistent with the notion that living arrangements are an important identifier of the differential risk of self‐injury and domestic violence during a pandemic. Young adults living alone had an increased risk of self‐injury; this is consistent with prior research on during‐pandemic hospitalized individuals with self‐injury who reported reduced opportunities for face‐to‐face contact with family members, friends, or partners, and a lack of social support (Hawton et al., 2021). Although we cannot tell from our data whether individuals who reported self‐injury during the pandemic already felt isolated before the pandemic, our findings identify young people who live alone as an important target population for interventions during a pandemic and perhaps also in postpandemic times.

The finding that young adults who have transitioned to cohabiting with a partner have a relatively high risk of domestic violence is in line with research conducted in prepandemic times showing that young cohabiting couples experience more violence than other couples (Berger, Wildsmith, Manlove, & Steward‐Streng, 2012). Here, we find this association in comparison with young adults living together with peers, which is consistent with our hypothesis that cohabiting with intimate relationship partners may be more challenging. However, the group difference could also indicate that those who choose to live with peers are, for example, more cooperative than those living with a partner.

Overall, the benefits of young adults gaining residential independence from their parents are not clear‐cut when it comes to maladaptive behavior and mental health. This is consistent with prior research (Copeland et al., 2015) that found an association between living arrangements without parents and reduced service use related to mental health problems. Our findings suggest that, during times of crisis and stress, living with parents, but also with other peers, may constitute a secure base with built‐in social support that can shield young adults from harm. However, there could also be selection effects whereby young adults with little support from parents may be more likely to move out of the parental home early (Seiffge‐Krenke, 2009).

### Prepandemic Behavioral Development

Our findings show considerable continuity between pre‐ and during‐pandemic self‐injury, underscoring the high risk of self‐injury maintenance once the behavior is initiated (Nock, 2009). Rules for social distancing and lockdown measures may not necessarily affect opportunities for self‐injury, which typically takes place in private (e.g., at home) anyway. This could also be a reason for the stable prevalence of self‐injury across the during‐pandemic assessments.

Associations of prepandemic violence perpetration with during‐pandemic domestic violence were relatively low, and our findings suggest that living arrangements and prepandemic anger may be better indices of an individual's risk of during‐pandemic domestic violence. However, the low association with prepandemic violence perpetration could, in part, be due to measurement: Our prepandemic assessment of violence measured the perpetration of violence committed in *or* outside of the home, whereas the during‐pandemic assessment was restricted to the home environment. An exploratory follow‐up analysis showed that during the lockdown, in April, those who had reported any violence perpetration prior to the pandemic had a four times higher risk of domestic violence perpetration than others (OR = 4.02, 95% CI = 1.61–10.06, *p* = .003), whereas the association was absent after the lockdown in September (OR = 1.54, 95% CI = 0.20–12.06, *p* = .681). These findings suggest that the social context of violence may have shifted from out‐of‐home to in‐the‐home during the lockdown and back to out‐of‐home thereafter in many cases. Further, stricter lockdown measures could reduce opportunities to act out aggressive impulses in nonviolent ways (e.g., due to the closure of sports clubs). Future research needs to further investigate how the rapid changes in public health, social, economic, and natural environments that occur during a pandemic (e.g., those due to governmental restrictions, reported case numbers of infections, season) affect young people's behavioral development.

### Pandemic‐Related Stressors, Indirect Effects through Emotions, and Coping Strategies

Consistent with our hypotheses, pandemic‐related stressor accumulation was associated with both self‐injury and domestic physical violence perpetration. Therefore, to avoid the additional burden of these behaviors, it is important that young adults who experience an accumulation of stressors during the pandemic receive adequate support. In line with previous research, we found that negative emotions, especially internalizing symptoms, are involved in the chain from stressor accumulation to both forms of harm (Farrington, 2018; Nock et al., 2006). These associations persisted over a period of at least five months (i.e., from April to September). Thus, strategies to regulate negative emotions are promising intervention targets for preventing self‐injury and domestic violence during the pandemic and potentially thereafter.

Notably, our models do not provide evidence of causality, and prior research found that the effects between self‐injury and, for example, depressive symptoms can be reciprocal (Zhu, Chen, & Su, 2020). Nevertheless, our results support the conclusion that effects operated from stressors to harm, given that all models, including the mediation models, controlled for prepandemic individual differences in self‐injury or violence, respectively, and emotions. Furthermore, an implicit timeline was incorporated in the data collection process (i.e., in April, the participants reported stressor accumulation since the beginning of the pandemic, which was ˜2 months ago, and self‐injury and domestic violence during the previous 2 weeks). Although there is some temporal overlap, this timeline suggests that stressor accumulation more likely occurred before the outcomes than vice versa. However, future research is needed to disentangle the potential reciprocal associations among stressors, emotions, and harm during a pandemic.

Our findings also show that coping strategies are relevant for young people's mental health during the pandemic, as was reported in other research (Zacher & Rudolph, 2021). Our findings suggest that strategies altering the ways in which young people perceive the pandemic (e.g., acceptance of the reality of the pandemic) are a promising target for interventions aimed at reducing the pandemic's psychological consequences. Indeed, acceptance as an emotion‐focused coping strategy (Litman, 2006) is an important target of therapeutic interventions in the realm of adolescent nonsuicidal self‐injury (Nock et al., 2007), which is often an attempt to regulate emotions. However, our measure of self‐injury did not distinguish between nonsuicidal and suicidal acts, and the roles of particular coping strategies for these different forms of self‐injury need to be investigated in future research.

Our findings on the role of acceptance of the reality of the pandemic also shed new light on the potential consequences of the various (political) movements based on denial (i.e., the opposite of acceptance) of the pandemic. Although these movements may create a sense of agency and group identity in some individuals, they can eventually pose a threat not only in terms of spreading the virus but also to young people's mental health.

### Limitations and Future Directions

First, our one‐item measure of self‐injury includes a range of relatively mild (e.g., hair pulling) to more severe forms of injury (e.g., cutting), excludes other forms of self‐harm (e.g., self‐poisoning), and does not distinguish between suicidal and nonsuicidal self‐injury. Coping strategies were also assessed with single items, which is not ideal. Second, socially undesirable and stigmatized behaviors, such as self‐injury and violence perpetration, are often underreported in self‐report surveys. However, the relatively high prevalence of any engagement in these behaviors in our sample suggests that underreporting was not a significant issue.

Third, our data do not allow for a distinction between age‐, cohort‐, and pandemic‐related changes in the prevalence of self‐injury and domestic violence. Fourth, the course of the COVID‐19 pandemic, governmental regulations, people's perceptions of these events, and people's socio‐economic resources and cultural backgrounds vary greatly across countries. It is uncertain to what extent our findings generalize to other regions. In addition, the psychological repercussions of the pandemic could be different and more severe in clinical and high‐risk samples compared with our community sample. Finally, our data were collected during the first months of the pandemic in Switzerland. Prolonged durations of the pandemic and its associated lockdowns are likely associated with different consequences for young adults' mental health.

## CONCLUSIONS

Experiences related to the COVID‐19 pandemic can contribute to the emergence and consolidation of individual differences in the risk of self‐injury and domestic physical violence perpetration during the transition to young adulthood. Our findings have important implications for researchers, politicians, and practitioners who aim to prevent social, mental and physical health, and financial costs following self‐injury and domestic violence during and after the pandemic. Screenings of young adults' risk of during‐pandemic self‐injury and domestic violence perpetration should consider prepandemic individual emotional and behavioral histories, especially of self‐injury, as well as current living arrangements, exposure to pandemic‐related stressors, and perceptions of the pandemic.

## Supporting information

Supplementary Material
