# Supplementary material for: Self‐Injury and Domestic Violence in Young Adults During the COVID‐19 Pandemic: Trajectories, Precursors, and Correlates
Source: J Res Adolesc. 2021 Jul 27;31(3):560–75. doi: 10.1111/jora.12659 (PMC8420612; doi:10.1111/jora.12659)
Supplement: Supplementary file 1 — Supplementary Material [file JORA-31--s001.docx]

**Online Supplement**

**Self-Injury and Domestic Violence in Young Adults During the COVID-19 Pandemic: Trajectories, Precursors, and Correlates**

*Annekatrin Steinhoff, Laura Bechtiger, Denis Ribeaud, Aja Louise Murray, Urs Hepp, Manuel Eisner, and Lilly Shanahan*

*Table S1.* List of Stressors During Lockdown and Pre-Pandemic Stressful Life Events (in Previous 3 Years, i.e., age 17-20)

| Measure | Items | Scale |
| --- | --- | --- |
| Cumulative stressors during pandemic | Have you experienced one of the following events in the context of the COVID-19 pandemic since the previous assessment?  Health-related events   - A close person tested positive for COVID-19 but was not admitted to hospital - A close person tested positive for COVID-19 and was admitted to hospital - A close person died due to COVID-19 - I had symptoms that could have been attributed to COVID-19 - I tested positive for COVID-19 but was not admitted to hospital - I tested positive for COVID-19 and admitted to hospital   What is your current occupational/educational/financial situation?   - I have had financial problems - My mother lost her job and is now unemployed - My father lost his job and is now unemployed - I lost my job and am now unemployed - My education program was cancelled - I cannot participate in my education program/go to work due to quarantine/self-isolation - I was furloughed - I am self-employed and lost customers, - I am self-employed and had to apply for government loans to help my business survive the pandemic | 1 = Yes  0 = No  Sum score  (possible range: 0-15) |
| Pre-pandemic stressful life events in past 3 years  (28 items) | Have you experienced one of the following events in the past three years?   - You moved in with a foster family or moved to a home. - You moved away from your parents and now live independently (alone, with a partner, or in a house-share). - You were hospitalized for several days because you were ill or because you had an accident. - You spent several days in a psychiatric clinic because of a psychological illness (e.g. depression). - Your father or your mother were hospitalized for several days because they were ill or because they had an accident. - Your sister or your brother was hospitalized for several days because s/he was ill or because s/he had an accident. - A good friend of yours was hospitalized for several days because s/he was ill or because s/he had an accident. - Your grandfather or grandmother died. - Your sister or brother died. - Your mother or father or another adult that cares for you at home died (e.g., your stepmother or your mother’s partner). - Another person you were close to died (e.g. a good friend, aunt, cousin, classmate). - Your parents divorced or separated and one of them moved away from home. - A new partner of your mother or of your father moved in with you (please also mark if you moved in with her/him). - A new brother or sister was born and now lives with you. - Your brother or sister moved away from home. - Your mother or father lost their job and became unemployed. - You had to repeat a grade. - You failed an important exam or probation period at school. - You couldn’t find an apprenticeship, even though you looked for one. - You quit your education or training or got fired from your apprenticeship. - You were reprimanded at school or had to go to the principal because of your behavior. - You were reported to the police and were questioned by them. - You were sentenced to prison. - You broke up with/were broken up with by a romantic partner (male/female). - You broke up with your best friend or he/she did not want to be friends with you anymore. - There was a burglary at your house. - You were forced to perform or endure sexual acts under serious threat or use of force. - You were victim of a violent crime and had to be medically treated as a consequence. | 1= Yes  0 = No  Sum score  (possible range: 0-28) |

*Table S2.* Items Used to Assess Coping Strategies (Carver, 1997; Shanahan et al., 2020).

| Coping strategy | Item |
| --- | --- |
| Acceptance | I have been accepting the reality that the corona crisis is real. |
| Positive Reappraisal/Reframing | I have been trying to find something good in the corona crisis (e.g., that that due to the crisis my relationship with a person that is important to me has become stronger). |
| Self-distraction | I have been distracting myself with other things to take my mind off things (e.g., watching TV or films, reading, daydreaming, sleeping, playing with my pet, online shopping, cooking, cleaning, etc.) |
| Emotional support seeking | I have been seeking emotional support from others (e.g., from family, friends). |
| Physical activity/exercise | I have exercised or engaged in physical activity. |
| Keeping in contact with family, friends, close others | I have been keeping in contact with my family, other close persons and friends. |
| Keeping daily routine | I have been trying to keep up a daily routine. |


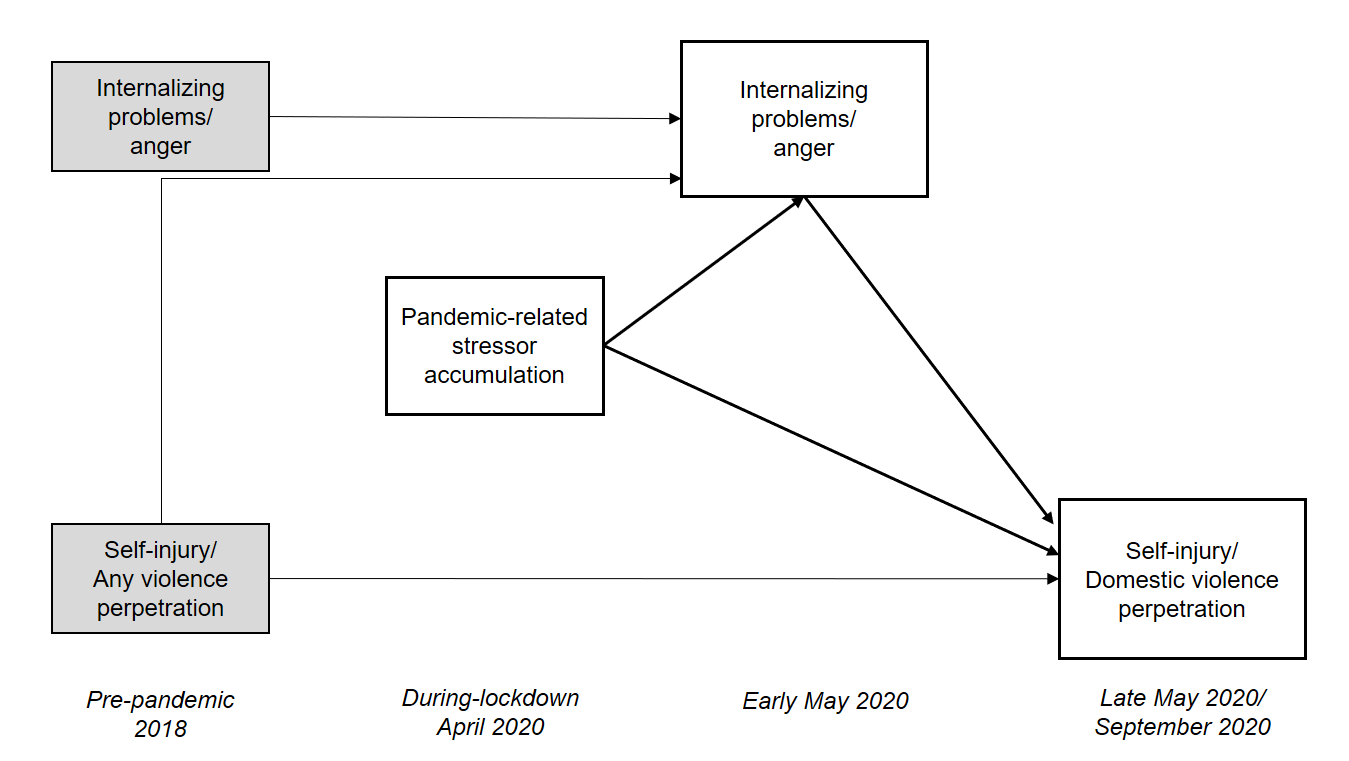


*Figure S1.* Illustration of Path Models Specified to Test Indirect Distal Effects From During-Lockdown Stressor Accumulation to Self-Injury/Domestic Physical Violence Perpetration, Respectively.

*Note.* The models were tested separately for internalizing symptoms and anger, and controlled for socio-demographics, pre-pandemic self-injury/violence perpetration, respectively, and during-pandemic living arrangements. Bold lines indicate the direct and indirect paths of main interest.


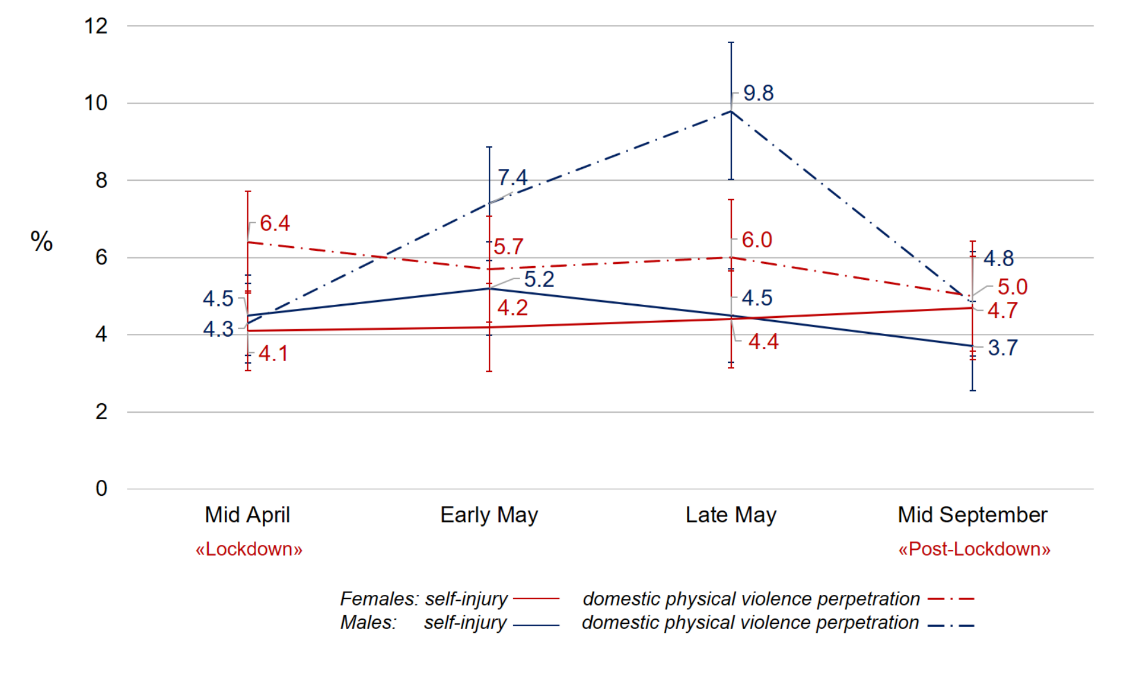


*Figure S2.* Sex-Specific Point Prevalence of Self-Injury and Domestic Physical Violence Perpetration Between April and September 2020.

*Note.* Prevalence of domestic violence perpetration refers to those not living alone.

**References**

Carver, C. S. (1997). You want to measure coping but your protocol's too long: consider the brief COPE. *International Journal of Behavioral Medicine, 4*(1), 92-100. doi:10.1207/s15327558ijbm0401_6

Shanahan, L., Steinhoff, A., Bechtiger, L., Murray, A. L., Nivette, A., Hepp, U., . . . Eisner, M. (2020). Emotional distress in young adults during the COVID-19 pandemic: evidence of risk and resilience from a longitudinal cohort study. *Psychological Medicine*, 1-10. doi:10.1017/s003329172000241x
